# Comparative physiochemical and transcriptomic analysis reveals the influences of cross-pollination on ovary and fruit development in pummelo (*Citrus maxima*)

**DOI:** 10.1038/s41598-023-46058-3

**Published:** 2023-11-04

**Authors:** Shaohua Wang, Chunrui Long, Hongming Liu, Li Pan, Shizao Yang, Jun Zhao, Yan Jiang, Xuejun Bei

**Affiliations:** 1 Institute of Tropical and Subtropical Cash Crops, Yunnan Academy of Agricultural Sciences, Baoshan, 678000 China; 2 Key Laboratory for Conservation and Utilization of Subtropical Bio-Resources, Education Department of Guangxi Zhuang Autonomous Region, Yulin Normal University, Yulin, 537000 China

**Keywords:** Molecular biology, Physiology, Plant sciences

## Abstract

‘Shuijingmiyou’ pummelo (SJ), one of the most popular fruits in Yunnan province of China, is of relatively low fruit shape (FS) quality. In this study, we compared the FS promoting effects of cross pollinations using pollens from seven pummelo varieties, and found that ‘Guanximiyou’ pummelo (GX) cross-pollination showed the best FS promoting effects on SJ fruits by shortening its fruit neck. To explore the underlying mechanism, physiochemical and transcriptomic differences between self- and cross-pollinated SJ ovaries (SJO and GXO) were investigated. Higher salicylic acid, gibberellin and indole acetic acid contents and superoxide dismutase, peroxidase and catalase activities, and lower polyphenol oxidase activity were determined in GXO compared with SJO. Enrichment analysis of the identified 578 differentially expressed genes (123 up-regulated and 455 down-regulated) in GXO showed that genes involved in solute transport, RNA biosynthesis, phytohormone action and cell wall organization were significantly enriched. The results obtained in this study will be helpful in understanding the influences of cross-pollination on pummelo ovary and fruit development, and can provide the basis for clarifying the underlying mechanism of cross-pollination improved fruit quality.

## Introduction

Fruit quality, including the internal quality and external quality, is an important purchasing-driving trait that has received great attentions to satisfy the requirements of consumers and market. Given the lack of rapid and cost-effective non-destructive detection method for internal quality^1^, external quality is usually considered as the first purchasing-driving trait. Fruit shape (FS), one of the most important external quality indexes, has been widely focused on because it is one of the first indicators of consumer concern and satisfaction and its high correlation with fruit yield and internal quality^2–5^. FS is usually evaluated by fruit length diameter (FLD), fruit diameter (FD) and fruit shape index (FSI, the ratio of FL to FD)^6^. Cross-pollination has been proven to be one of the most effective and reliable. This method has been successfully utilized in FS improvement of many fruits, such as apple^7^, guava^8^, kiwifruit^9^, lychee^10^.

Pollination significantly affects the ovary and fruit development. However, for many flowering plants, self-compatibility and successive self-pollinations will lead to species decline and genetic diversity reduction^11–14^. Therefore, many fruit crops, such as *strawberry*^15^, *Citrus*^16^, *Pyrus pyrifolia*^17^, *apricot*^18^, *Prunus salicina*^19^, *P. pseudocerasus*^20^, *Eriobotrya japonica*^21^ and *apple*^22^, evolved and preferred the self-incompatibility (SI) strategy. The pollen tubes of SI plants cannot enter their own style, resulting in the failure of fertilization. However, exogenous pollens can germinate more easily germinate on the stigma, enter the ovary to complete fertilization, and ultimately trigger the ovary/fruit development^23^. Evidence has shown that pollination greatly influences the fruit development by regulating the expression of genes involved in auxin, cytokinin, and gibberellin biosynthesis and signaling pathways and by mediating the hormone dynamics^24–27^. Studies have also shown that self-pollination will induce the accumulations of reactive oxygen species (ROS), This inhibits the pollen germination and elongation on style, and resulting in the programmed cell death (PCD)^28^. however, cross-pollination can suppress pollination-induced ROS accumulation, facilitating the accomplishment of fertilization and ovary development^29^.

‘Shuijingmiyou’ pummelo (*Citrus maxima.* ‘Shuimingmiyou’, SJ) is one of the most popular fruit crops in tropic and subtropic areas in Yunnan province of China. The fruits of SJ have pleasant flavor and taste. But its FS shows long fruit neck and high rate of fruit-neck length/fruit longitudinal diameter (RF). Local consumers dislike this appearance quality, and much prefers pear-shaped fruit of SJ with short fruit neck length. Therefore, The FS improvement of SJ is very necessary to meet the ever-increasing awareness and demand for high appearance quality-assured fruit^2^. In our previous study, we demonstrated that cross-pollination could significantly increase the fruit-setting rate, flesh weight, edible rate, the contents of both soluble solids and titratable acid of SJ fruits^30^. Moreover, cross-pollination using exogenous pollens produced pear-shaped SJ fruits, whose FS quality was greatly improved. However, the underlying mechanism is still not clear. In this study, cross-pollination experiments using exogenous pollens from seven pummelo varieties (including Guanximiyou pummelo (GX), Myanmar pummelo (MP), Sour pummelo (SP), Acid-less pummelo (AP), Shatian pummelo (STP), Wanbai pummelo (WP) and HB pummelo (HB), were performed on SJ with SJ self-pollination as a control, given the FS-improving and inner qualities effect of exogenous pollens. and the GX pollen showed the best FS promoting effects on SJ fruits. We further observed the development of GX and SJ pollen grains in the SJ stigma. We determined the enzyme activities of superoxide dismutase (SOD), polyphenol oxidase (PPO), phenylalanine ammonia-lyase (PAL), catalase (CAT) and peroxidase (POD), contents of H_2_O_2_, malondialdehyde (MDA) and proline (Pro), and the contents of indole acetic acid (IAA), jasmonic acid(JA), salicylic acid (SA), gibberellin (GA) and abscisic acid (ABA) in GX pollinated SJ ovary (GXO) and SJ pollinated SJ ovary (SJO) to explore the mechanism underlying this phenomenon. The RNA-seq technique has been successfully applied in exploring the transcriptome differences between self-pollinated and cross-pollinated stigma^31^ and fruits^32,33^. We observed significant differential expression of genes involved in plant hormone signal transduction, glutathione metabolism, ribosome and oxidative phosphorylation and so on, indicating that these genes contributed to the fruit development by regulating complicated pathways. A comparative transcriptomic analysis between GXO and SJO was also performed to uncover the possible molecular mechanism involved in the GX cross pollination improved SJ fruit shape. Our study helps to understand the mechanism for the cross-pollination improved SJ fruit shape, and provides basis for the quality improvement of pummelo fruit.

## Materials and methods

### Pollens collection and pollination

The pollens of SJ, GX, MP, SP, AP, STP, WP and HB were collected in Institute of Tropical and Subtropical Cash Crops, Yunnan Academy of Agricultural Sciences (Ruili, Dehong Dai and Jingpo Autonomous Prefecture, Yunnan province of China. Longitude, N97°43, 49.68, E24°54, 52.95) from May 2015 to early March 2016, and germination rate of all pollens in vitro was more than 80% by Liang ^16^. After removing the stamens, the flowers of SJ plants were pollinated respectively using SJ, GX, MY, SP, AP, STP, WP and HB pollens at the full-bloom stage in March 2016. At least 150 SJ flowers were pollinated for each treatment.

### Measurement of fruit shape-related parameters

Fruit shape related parameters (supplemental Figure S1), including signal fruit weight (SFW), fruit longitudinal diameter (FLD), fruit width (FW) and fruit-neck length (FNL), were measured in October 2016. Also, the fruit shape index (FSI, fruit longitudinal diameter/fruit width) and the fruit-neck length/fruit longitudinal diameter (RF) rate were calculated. All the results are shown as average ± SD (standard deviation) of nine fruits.

### Pollen germination observation

To investigate the influences of GX cross-pollination and self-pollination on the fertilization and ovary development. The pollens of GX and SJ were pollinated to SJ stigmas in March 2017, respectively. At six days post pollination, ovaries were collected and divided into two groups. Ovaries of the first group were immediately fixed in formalin/acetic acid/alcohol (FAA). After fixing, the pistils were stained with the aniline blue fluorochrome according to the method described by Lin et al.^34^. Ovaries of the other group were collected in 50 mL tubes, immediately frozen in liquid nitrogen, and stored at − 80 °C for further use.

### Determination of plant hormones contents, antioxidant enzymes activities and antioxidant related molecules contents

Self-pollination and cross-pollination ovaries (Fruitlet, sixth day after pollination) were taken as experimental material in this paper. The contents of IAA, GA, ABA, SA, and JA in GXO and SJO were measured using enzyme-linked immunosorbent assays (ELISA) methods^35^. The SOD, POD, CAT, POD and PAL enzyme activities and the contents of H_2_O_2_, MDA, and proline in GXO and SJO were determined by using corresponding commercial kits (Comin, Suzhou, China) according to the manufacturer’s instructions. Three biological replications were performed for each group.

### Total RNA isolation and RNA sequencing

In the paper, self-pollination and cross-pollination ovaries (Fruitlet, sixth day after pollination) were used as research material. Total RNA from the GXO and SJO was extracted according Trizol reagent (Life technologies, USA). The total RNA was treated with RNase-free DNase I (TaKaRa, Dalian, China) to remove the contaminated DNA. The quality and quantity of each RNA sample were assessed by 1% agarose gel and NanoDrop 2000c UV–Vis Spectrophotometer (Thermo Scientific, Wilmington, DE, USA). The mRNA was isolated by NEBNext Poly (A) mRNA Magnetic Isolation Module (NEB). The enriched and purified mRNA was broken into approximately 200nt short RNA inserts, which were used to synthesize the first-strand cDNA and the second cDNA. The double-stranded cDNA were used to perform end-repair/dA-tail and adaptor ligation. The suitable fragments were isolated by AgencourtAMPure XP beads (Beckman Coulter, Inc.), and enriched by PCR amplification. Finally, the constructed 6 cDNA libraries of the samples were sequenced on a flow cell using an Illumina HiSeq™ 2000 sequencing platform^36^. All the transcriptome data were deposited in NCBI (https://www.ncbi.nlm.nih.gov/) with BioProject ID of PRJNA795605 and BioSample accession number of SAMN24737524.

### Identification and analysis of differentially expressed genes

After removing the reads containing adapter or ploy-N and the low-quality reads, clean reads were obtained and were aligned to the genome of *C. grandis* (L.) Osbeck cv. ‘Wanbaiyou’ genome (http://citrus.hzau.edu.cn/download.php) using HISAT^37^. Reads number was normalized into fragments per kilobase of transcript sequence per millions base pairs sequenced (FPKM) and were then used to assess the relative gene expression levels. Differentially expressed genes (DEGs) between GXO and SJO were identified using DESeq R package with *p*-values < 0.05 as a criterion^38^. Gene ontology (GO) and Kyoto Encyclopedia of genes and genomes (KEGG) enrichment analysis of DEGs were performed using corrected *p*-values < 0.05 as a criterion^39–41^.

All the *C. grandis* (L.) Osbeck cv. ‘Wanbaiyou’ CDS sequences were submitted to Mercator v.3.6 (https://plabipd.de/portal/mercator-sequence-annotation) to obtain the mapping file used for MapMan analysis^42^. Pathway enrichment analysis was performed using PageMan embedded in MapMan^43^.

### Quantitative real-time PCR analysis

The expression analysis of six representative genes, including *putative indole-3-acetic acid-amido synthetase GH3.9* (*GH3.9*, cg5g037090), *ent-copalyl diphosphate synthase* (*CPS*, cg5g035420), *MYB-related protein 306* (*MYB306*, cg6g008620), *WRKY transcription factor 41* (*WRKY41*, cg5g001540), *cellulose synthase-like protein E6* (*CSPE6*, cg4g013930) and *xyloglucan endotransglucosylase/hydrolase protein 22* (*XYL22*, cg4g022120), were performed on a LightCycler480II instrument (Roche, Switzerland) using SYBR Green real-time PCR Master Mix (Toyobo, Osaka, Japan) according to Zhong et. al^44^. The quantitative Real-Time PCR (qRT-PCR) condition was set as: pre-denaturation at 95℃ for 1 min, followed by 40 cycles of 95 °C for 5 s, 60 °C for 10 s and 72 °C for 15 s. The Actin gene was used as the endogenous control^44^, and the relative expression levels of the genes was calculated using the 2^−ΔΔCt^ method^45^. The information on all the primers used in this study is listed in supplemental Table S3.

### Data analysis

All the data were processed and calculated using Excel2010 (Microsoft, Redmond, Washington, USA). Data difference significance was calculated using t-test conducted in SPSS version 19 (IBM, Chicago, IL, USA)^46^.

### Ethics approval and consent to participate

All experimental research was conducted in accordance with relevant institutional, national, and international guidelines and legislation. Plant samples used in the study were not collected from national park or natural reserve. ‘Shuijingmiyou’ pummelo was kept at Institute of Tropical and Subtropical Cash Crops, Yunnan Academy of Agricultural Sciences. We declared that the permissions were obtained for the collection of plant material (from Institute of Tropical and Subtropical Cash Crops, Yunnan Academy of Agricultural Sciences), and the permission was obtained from Institute of Tropical and Subtropical Cash Crops. We confirm that this complies with national guidelines and no formal ethics approval was required in this particular case.

## Results

### The influences of cross-pollination on the fruit shape indexes of SJ pummelo

Pollens of SJ, GX, MP, SP, AP, STP, WP and HB were collected and used for SJ pollination. Fruit shape indexes were determined and calculated, including SFW, FLD, FW, FNL, FSI and RF (Table 1). Cross-pollination significantly influenced the shape of SJ fruits. Compared to the self-pollinated SJ fruits, the SFWs of the cross-pollinated SJ fruits all increased. The SFW of HB pollinated SJ fruits was the largest, accounting for about 1.52-fold that of self-pollinated SJ fruits. The SFW of MP, STP, AP, GX and WP pollinated fruits were 1.35-, 1.35-, 1.30-, 1.27- and 1.21-fold of the control SJ fruits, respectively. All the SFWs of cross-pollinated SJ fruits except SP were found to be significantly higher than that of the self-pollinated controls.

**Table 1:** Influences of cross pollination on the fruit shape indexes of SJ fruits.

Species	SFW/g	FLD/cm	FW/cm	FNL/cm	RF/%	FSI
SJ	1373.53 ± 35.33	18.40 ± 0.28	16.43 ± 0.16	5.30 ± 0.07	28.80 ± 0.15	1.12 ± 0.015
GX	1739.05 ± 69.71**	18.78 ± 0.46	17.28 ± 0.35*	3.87 ± 0.03**	20.61 ± 0.11**	1.08 ± 0.016
SP	1448.31 ± 50.68	18.33 ± 0.81	16.53 ± 0.78	4.23 ± 0.71*	23.07 ± 0.09*	1.10 ± 0.008
AP	1784.78 ± 54.29**	18.04 ± 0.31	17.37 ± 0.28*	4.06 ± 0.19*	22.5 ± 0.21*	1.03 ± 0.016*
MP	1852.92 ± 73.85**	18.77 ± 0.48	17.80 ± 0.27*	4.34 ± 0.21*	23.12 ± 0.34*	1.04 ± 0.020*
STP	1847.83 ± 76.64**	18.94 ± 0.28	17.88 ± 0.63*	4.55 ± 0.22*	24.02 ± 0.36*	1.09 ± 0.015
WP	1657.66 ± 39.16**	17.21 ± 0.84*	17.16 ± 0.31	3.96 ± 0.30**	23.01 ± 0.21*	1.05 ± 0.023*
HB	2092.00 ± 92.25**	20.60 ± 0.57*	18.96 ± 0.48**	6.43 ± 0.11**	31.21 ± 0.15*	1.08 ± 0.007

* and ** indicates significant changes at *p* < 0.05 and* p* < 0.01 level, respectively.

The FLDs of HB, STP, GX and MP cross-pollinated SJ fruits were higher than those of self-pollinated fruits, accounting for about 1.12-, 1.03-, 1.02- and 1.02-fold of the self-pollinated controls. The FLD of HB pollinated SJ fruits was significantly higher than controls. However, the FLD of WP pollinated SJ fruits was markedly lower than that of the controls. HB pollination significantly improved and STP, MP, AP and GX pollination significantly increased the FW of SJ fruits compared to self-pollinated SJ fruits.

HB pollination significantly increased the FNL of SJ fruits compared with the self-pollinated controls (Table1). However, cross-pollination using pollens of other pummelo cultivars all considerably reduced the FNL. Notably, GX and WP pollination significantly reduced the FNL values of SJ fruits (Table 1), accounting for about 73.02% and 74.72% of the self-pollinated controls, respectively.

HB pollination significantly increased the RF of SJ fruits compared with the self-pollinated controls (Table 1). However, cross-pollination using pollens from other pummelo species all significantly reduced the RF. Notably, GX pollination significantly reduced the RF values of SJ fruits (Table 1), accounting for only about 71.56% of the self-pollinated controls. Cross-pollination using different pollens all reduced the FSI of SJ fruits. And AP, MP, and WP pollination showed significantly high suppression effects on the FSI of SJ fruits.

**Figure 1:**
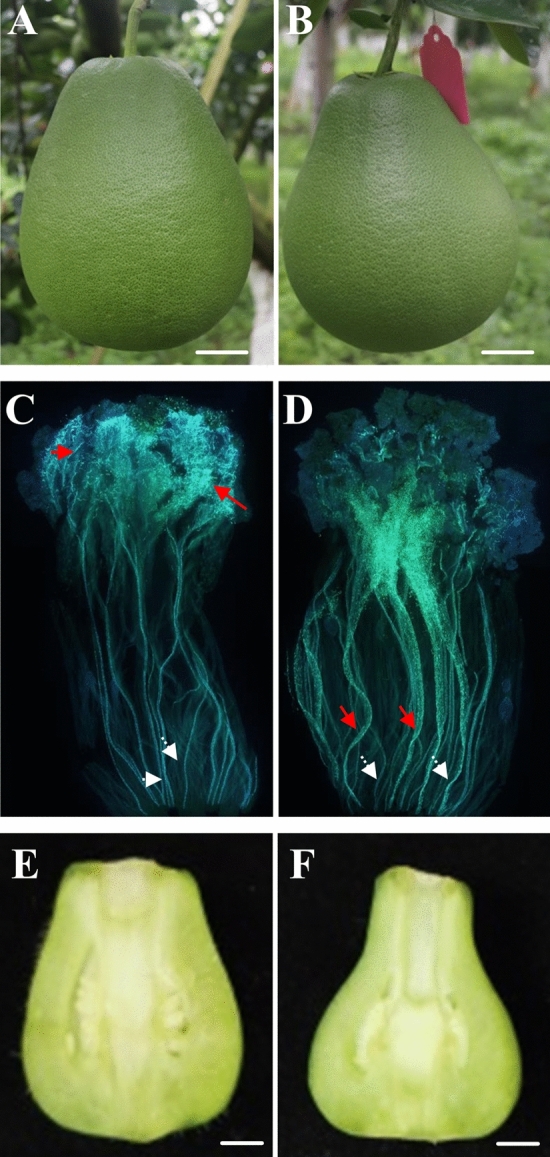
Influences of GX cross-pollination on the shape of SJ fruits and ovaries. (**A**) and (**B**): self-pollinated and GX cross-pollinated SJ fruit at 100 days post flowering; (**C**) and (**D**): SJ and GX pollen elongation in SJ ovary at 6 days post pollination; E and F: morphology of self-pollinated and GX cross-pollinated ovary at 6 days post pollination. Red and white arrows in (**C**) and (**D**) represent pollen tubes and vascular bundles, respectively. The scale bar in (**A**) and (**B**) is 2 cm, and the scale bar in (**E** and **F**) is 1 mm.

HB cross-pollination significantly increased the fruit size of SJ but significantly elongated its fruit neck, making the external fruit quality even worse. However, GX cross-pollination increased the fruit size and shortened the fruit neck of SJ fruits. Therefore, GX cross-pollination was considered as the best cross-pollination method for the FS improvement of SJ (Fig. 1A and 1B, Table1).

### Comparative analysis results of GX and SJ pollen germination in SJ stigma, contents of phytohormones, activities of antioxidant related enzymes and contents of antioxidant related molecules in GXO and SJO

GX and SJ pollen germination in SJ stigma was observed using aniline blue fluorochrome staining method^43^. At six days post pollination, both GX pollens and SJ pollens germinated in the SJ stigma, and GX pollen tubes successfully reached the SJ ovary (Fig. 1C,D). The SJ pollens, however, mainly stayed on the stigma and no pollen tube was observed in the SJ ovary, indicating that the SJ pollen elongation was restrained. Moreover, the SJ ovary morphology changed considerably due to GX cross-pollination at six days post pollination (Fig. 1E,F).

**Figure 2:**
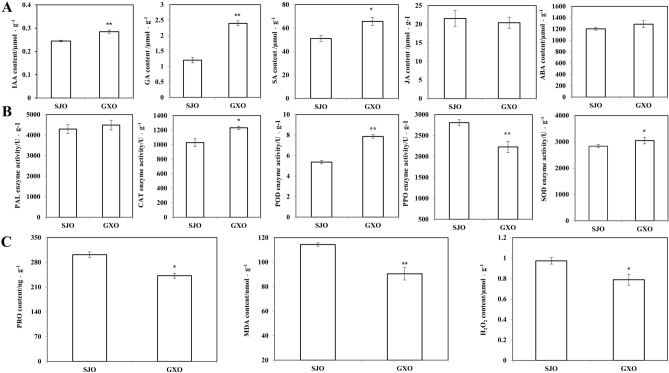
Influences of GX cross-pollination on the contents of phytohormone, activities of antioxidant-related enzymes, and contents of antioxidant-related molecules of SJ ovaries. (**A**): contents of IAA, GA, ABA, JA, and SA in SJO and GXO; (**B**): enzyme activities of POD, CAT, PAL, PPO and SOD in SJO and GXO; (**C**): the contents of Pro, MDA, and H2O2 in SJO and GXO. * and ** indicates significant difference at *p*-value < 0.05 level and significant difference at *p*-value < 0.01 level, respectively.

To investigate the influences of cross-pollination on phytohormone accumulation in SJ ovaries, the JA, SA, IAA, GA, and ABA contents in GXO and SJO were determined. The IAA and GA content in GXO was significantly higher than that in SJO (Fig. 2A), accounting for approximately 1.16- and 1.99-fold of that in SJO, respectively. The SA content in GXO significantly higher than in SJO. No significant differences in JA and ABA contents were found between GXO and SJO.

Further, we determined the enzyme activities of SOD, POD, PAL, PPO and CAT in both GXO and SJO. The PPO activity in GXO was found to be significantly lower than that in SJO. However, the POD activity in GXO was significantly higher than that in GXO, and the SOD and CAT activities in GXO were both significantly higher than that in SJO (Fig. 2B). No significant difference in PAL activity was found between GXO and SJO.

MDA, H_2_O_2_ and Pro contents in GXO and SJO were also measured. Again, the MDA content in GXO was significantly lower than that in SJO, and the H_2_O_2_ and Pro contents in GXO were both significantly lower than that in SJO (Fig. 2C).

### Overview of the GXO and SJO transcriptome data

**Figure 3:**
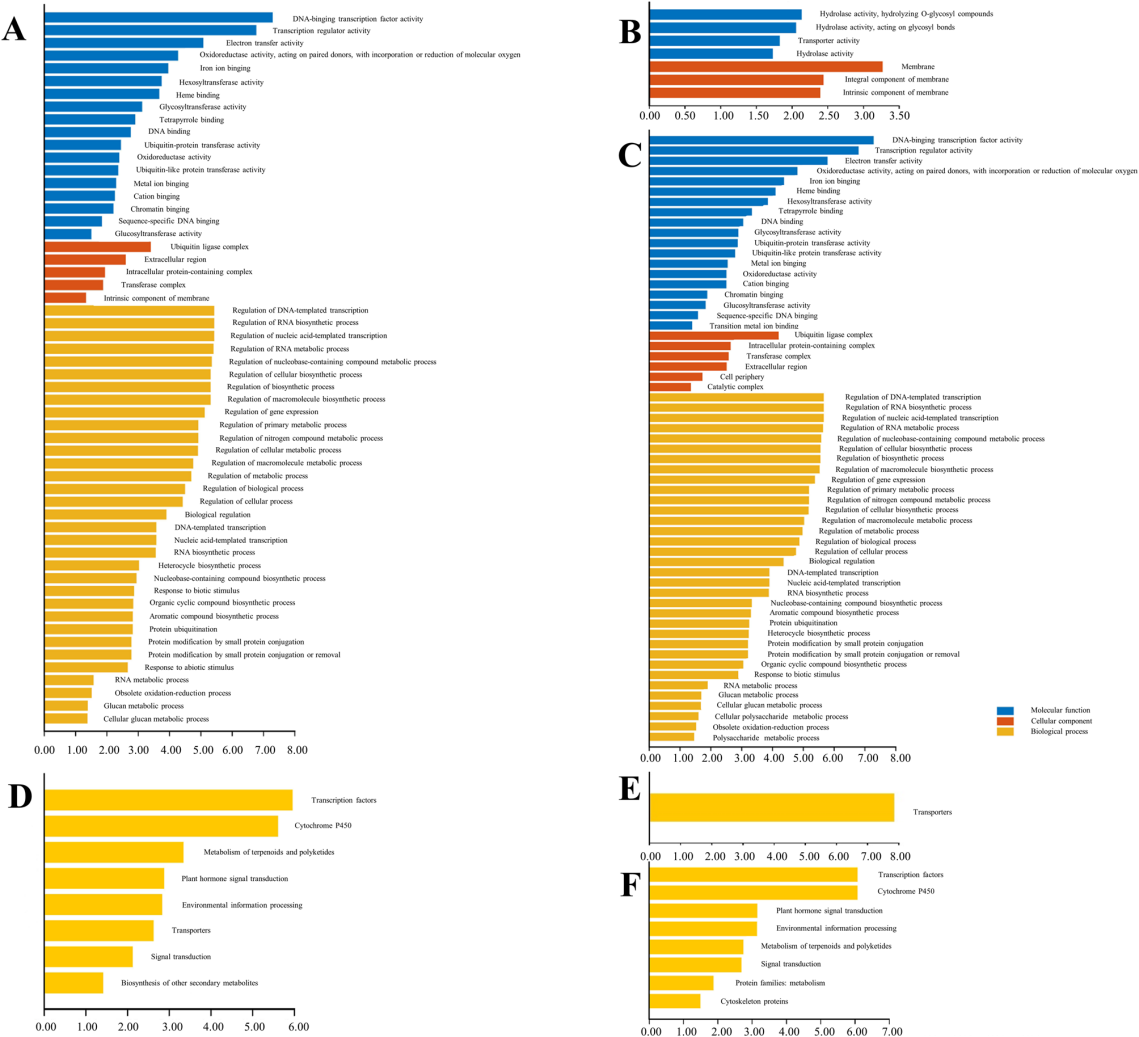
GO and KEGG enrichment analysis results for DEGs identified between GXO and SJO. (**A**): GO enrichment analysis results of all DEGs; (**B**): GO enrichment analysis results of up-regulated DEGs; (**C**): GO enrichment analysis results of down-regulated DEGs. (**D**): KEGG pathway enrichment analysis results of all DEGs; (**E**): KEGG pathway enrichment analysis results of up-regulated DEGs; (**F**): KEGG pathway enrichment analysis results of down-regulated DEGs.

RNA-seq was applied to the transcriptome profiling of GXO and SJO with three replications. After removing the low-quality reads, about 56,711,250 ~ 67,434,222 clean reads were obtained from each library with a mapping ratio ranging from 82.14% to 84.75% against the *C. grandis* (L.) Osbeck cv. ‘Wanbaiyou’ genome. More than 80% of the squences of all the six libraries were uniquely mapped to the genome (Supplemental Table S2). Totally, we identified 18,320 expressed genes from all the six libraries. A total of 578 DEGs were identified between the GXO and SJO using |log2(fold change)|> 1.5 and corrected *p*-value < 0.05 as criteria. Among these DEGs, 123 were upregulated and 455 were downregulated in GXO compared with SJO. GO analysis of all the DEGs revealed the significant enrichment of 56 GO terms, including 18 molecular functions (MF) related, five cellular component (CC) related, and 33 biological process (BP) related GO terms (Fig. 3A). GO analysis of the 123 upregulated genes in GXO showed that genes involved in hydrolyzing O-glycosyl compounds (GO:0,004,553), hydrolase activity, acting on glycosyl bonds (GO:0,016,798), transporter activity (GO:0,005,215), hydrolase activity (GO:0,016,787), membrane (GO:0,016,020), integral component of membrane (GO:0,016,021) and intrinsic component of membrane (GO:0,031,224) were significantly enriched (Fig. 3B). GO analysis of the 454 downregulated genes in GXO showed that genes involved in 49 significantly enriched GO terms, including 28 BP, 15 MF and six CC related GO terms (Fig. 3C).

KEGG enrichment analysis of all the DEGs showed that these genes were significantly enriched in eight pathways (Fig. 3D), including “Transcription factors”, “Cytochrome P450”, “Metabolism of terpenoids and polyketides”, “Plant hormone signal transduction”, “Environmental information processing”, “Transporters”, “Signal transduction”, and “Biosynthesis of other secondary metabolites”. Additionally, the upregulated DEGs were significantly enriched in “Transporters” pathway (Fig. 3E). The downregulated DEGs were significantly enriched in eight pathways, including “Transcription factors”, “Cytochrome P450”, “Environmental information processing”, “Metabolism of terpenoids and polyketides”, “Signal transduction”, “Protein families: mtabolism”, and “Cytoskeleton” (Fig. 3F).

### PageMan enrichment analysis of DEGs

We applied PageMan to analyze the enrichment of all DEGs identified between GXO and SJO. These genes were significantly enriched in “solute transport”, “RNA biosynthesis”, “phytohormone action”, and “cell wall organization” pathways (Table 2 and supplemental Table S3). Among the DEGs, 32 DEGs (14 up-regulated and 18 down-regulated) were categorized into the “solute transport” pathway, including four “primary active transport”-, twenty-six “carrier-mediated transport”- and two “channels”-related genes. Among the “primary active transport”- related DEGs, one *cadmium/zinc-transporting ATPase HMA2* gene (cg4g021370) and one *ABC transporter G family member 14* gene (*ABCG14*, cg6g020410) were significantly upregulated in GXO with a fold change of 1.78-fold and 1.53-fold, respectively. Conversely, two *ABC transporter G family member* genes (cg3g019410 and cg7g015090) were downregulated in GXO. Of the 26 “carrier-mediated transport”-related DEGs, one *UDP-rhamnose/UDP-galactose transporter 2* gene (*URGT2*, cg6g012450) and four *WAT1-related protein* genes (*WAT1*, cg6g012300, cg3g016360, cg6g017490, and cg3g007150) were significantly upregulated in GXO with fold change of 1.73-fold, 2.17-fold, 1.89-fold, 2.55-fold and 3.15-fold change, respectively. In addition, three sugar transport protein genes [*Hexose carrier protein HEX6* (cg9g023370), *sugar transport protein 14* (*STP14*, cg1g012330) and *sugar transport protein 10* (*STP10*, cg1g013990)] showed a significant difference between SJO and GXO. Two sugar efflux transporter genes [*bidirectional sugar transporter SWEET3b* (cg9g028280, upregulated about 1.97-fold in GXO) and *SWEET13* (cg7g010660, downregulated about 2.89-fold in GXO)] had adverse expression change patterns in GXO compared to SJO. Besides, one gene (cg9g008980) encoding *phosphate transporter PHO1* and two genes (cg9g029160 and cg9g029170) encoding *zinc transporter 1* were found to be up-regulated in GXO for 1.95-fold, 1.68-fold, 1.84-fold, respectively. Moreover, one gene (cg3g018280) encoding *vacuolar iron transporter homolog 3* was found to be GXO-specific.

**Table 2:** Significantly enriched pathways of DEGs identified between GXO and SJO using PageMan.

Bin	Name	Elements	*p*-value
24	Solute transport	32	8.90E-4
24.2.1	Solute transport. Carrier-mediated transport. DMT superfamily	5	0.001971
24.2	Solute transport. Carrier-mediated transport	26	0.002189
50.3.2	Enzyme classification.EC_3 hydrolases. EC_3.2 glycosylase	4	0.002793
24.2.1.5	Solute transport. Carrier-mediated transport. DMT superfamily. solute transporter *(UmamiT)	4	0.004034
21.3	Cell wall organisation. Pectin	4	0.009107
15	RNA biosynthesis	68	0.009419
15.5	RNA biosynthesis. Transcriptional regulation	67	0.014495
11	Phytohormone action	22	0.017702
21	Cell wall organisation	6	0.018434
15.5.9	RNA biosynthesis. Transcriptional regulation.AP2/ERF transcription factor superfamily	15	0.046541
15.5.9.1	RNA biosynthesis. Transcriptional regulation.AP2/ERF transcription factor superfamily. Transcription factor (ERF) activities	15	0.046542
11.6.1	Phytohormone action. Gibberellin. biosynthesis	2	0.047600

Similar to the KEGG enrichment analysis results, 22 DEGs were categorized into the “Phytohormone action” pathway by MapMan annotation. Four, three, two and three genes participated in the metabolism of ABA, auxin (IAA), cytokinin (CK) and gibberellin (GA), respectively. One gene encoding *putative indole-3-acetic acid-amido synthetase GH3.9* (*GH3.9*, cg5g037090) was significantly upregulated by about 2.12-fold in GXO. The CK metabolism related *ABCG14* gene was significantly up-regulated for about 1.53-fold in GXO. Two GA biosynthesis-related genes, *gibberellin 20 oxidase* (*GA20*, cg9g026010) and *Ent-copalyl diphosphate* (*CPS*, also named *GA requiring 1* (*GA1*), cg5g035420) were significantly upregulated in GXO by 2.60-fold and 1.88-fold, respectively. Interestingly, all the four ABA metabolism-related DEGs were down-regulated in GXO.

Six “Cell wall organization”-related DEGs were significantly upregulated in GXO. Among them, two genes (cg9g025270 and cg9g025290) encoding *21 kDa proteins* were upregulated for 1.50-fold and 2.14-fold, respectively. One gene (cg2g044560) encoding *fn3 like domain containing protein* was found to be specially expressed in GXO. One gene (cg2g013050) encoding *probable pectate lyase P18* was up-regulated about 1.69-fold in GXO. One gene (cg2g034080) encoding *putative fasciclin-like arabinogalactan protein 20* was upregulated about 1.87-fold. Finally, one gene (cg1g027350) encoding *alkane hydroxylase MAH1* was downregulated about 2.28-fold in GXO.

The 68 “RNA biosynthesis”- related DEGs included 67 “transcriptional regulation” genes and one “RNA polymerase I-dependent transcription initiation factor” related gene. Most of the “transcriptional regulation”-related DEGs were downregulated in GXO compared to SJO.

### Quantitative real time PCR result

**Figure 4:**
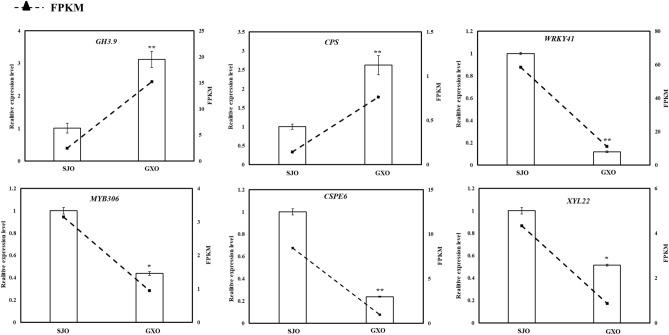
Quantitative real-time PCR validation results of six differentially expressed genes. *GH3.9*: *putative indole-3-acetic acid-amido synthetase GH3.9*, *CPS*: *ent-copalyl diphosphate synthase*, *MYB306*: *MYB-related protein 306*, *WRKY41*: *WRKY transcription factor 41*, *CSPE6*: *cellulose synthase-like protein E6*, *XYL22*: *xyloglucan endotransglucosylase/hydrolase protein 22*. * and ** indicates significant difference at *p*-value < 0.05 level and significant difference at *p*-value < 0.01 level, respectively.

The expression of *putative indole-3-acetic acid-amido synthetase GH3.9* (*GH 3.9*, cg5g037090) and *ent-copalyl diphosphate synthase* (*CPS*, cg5g035420) was upregulated in GXO. On the other hand, the expression of *MYB-related protein 306* (*MYB306*, cg6g008620), *WRKY transcription factor 41* (*WRKY41*, cg5g001540), *cellulose synthase-like protein E6* (*CSPE6*, cg4g013930) and *xyloglucan endotransglucosylase/hydrolase protein 22* (*XYL22*, cg4g022120) was downregulated in GXO. Consistence with our transcriptome data, we identified the same change patterns of all the selected genes using qRT-PCR (Fig. 4), indicating that our transcriptome data was believable.

## Discussion

Self-incompatibility (SI) is common for many Citrus species^16,47,48^. For example, ‘Shuijingmiyou’ pummelo, one of the most popular pummelo variety in Yunnan province of China, is of strong SI ability. For plants with SI ability, self-pollen can hardly germinate on their own style or enter into their ovaries. Still, exogenous pollens can germinate and access the ovary successfully in a relatively short time^49–51^. Our present study consistently found that GX pollens successfully entered the SJ ovary at six days post pollination. Moreover, GX cross-pollination showed the best FS promoting effects on SJ fruits mainly through shortening the fruit neck. Determinations of many physiochemical parameters and comparative transcriptomic analysis were performed to reveal the underlying mechanism of the GX cross pollination promoting effects on SJ fruit shape.

### Hormone metabolism differed significantly between GXO and SJO

Plant hormone homeostasis greatly influences almost all the plant growth and development processes^32^. Exogenous auxin treatment can promote pollen germination, pollen tube growth, and elevate endogenous auxin accumulation^52^. The roles of auxin biosynthesis-related genes in the SI response and in regulating ovary development have also been uncovered^53,54^. In this study, the IAA content in GXO was found to be about 1.16-fold of that in SJO, and a *GH3.9* gene showed much higher expression in GXO than in SJO, and its up-regulation in GXO was proved by qRT-PCR.

GAs, tetracyclic diterpenoid hormones, control diverse aspects of plant growth and development, such as root and shoot elongation, seed germination, flower transition, and fruit set and expansion^55–58^. In our present study, the GA content in GXO was about two-fold of that in the SJO, suggesting that cross pollination significantly increased the GA biosynthesis in the SJ ovary. Furthermore, *GA20*, an essential gene in GAs biosynthesis, contributed significantly to GAs biosynthesis^49,59,60^. In this study, a *GA20* gene was found to be up-regulated by more than 2.5-fold in GXO compared to SJO, and the expression of a *CPS* gene in GXO was about 1.88-fold of that in SJO, and both their expression patterns were verified by qRT-PCR.

SA is generally considered as an important stress-related phytohormone^61,62^. Furthermore, its external application is thought to be help to cross-pollination^33^. Consistently, in our study, the SA content in GXO was significantly higher than in SJO, suggesting that the SA accumulation is helpful for successful pollination and fertilization.

ABA contributed significantly to regulating the differentiation of floral organs^63^, fruit ripening^64^, reproduction process^65,66^, and ovary development^67^. ABA accumulation was significantly repressed by GA_4+7_ or pollination in peaches^68^. GA_4+7_and pollination repressed the ABA biosynthetic genes of the 9-cis-epoxycarotenoid dioxygenase family. In tomato, ABA levels decreased after pollination, an essential regulator of ABA biosynthesis in tomato is *9-cis-epoxy-carotenoid dioxygenase* (*LeNCED1*), whose mRNA level in ovaries is reduced after pollination^69^. Our study identified four ABA biosynthesis related-genes whose expression level were down-regulated in SJO, including two *NCED* genes and two *ABA 8'-hydroxylase* genes.

Ethylene plays a positive role in the SI-induced PCD. Applying aninooxyacetic acid (AOA, an inhibitor of 1-aminocyclopropane-1-carboxilic acid biosynthesis) suppressed ethylene trigged^60^, indicating that the inhibition of ethylene was helpful for the successful fertilization. Consistently, almost all (14 out of 15) the ethylene metabolism-related DEGs were downregulated in GXO in our study.

Cross-pollination using GX pollens strengthened the IAA, GA and SA biosynthesis, inhibited the ABA and ethylene accumulation in the SJ ovary, which ultimately facilitated the pollen germination, pollen tube development and ovary development.

### Cross pollination suppressed the ROS accumulation and significantly influenced the cell wall metabolism in the SJ ovary

Self-pollination has been reported induce the accumulation of ROS^28^, At the same time, cross pollination significantly suppressed the ROS accumulation, which facilitate the successful fertilization and ovary development^29^. Consistently, the H_2_O_2_ content in GXO was substantially lower than that in SJO. On the other hand, the SOD, CAT and POD activities in GXO were all higher than that in SJO, which will salvage ROS. Moreover, the contents of MDA and Pro in GXO were much lower, suggesting that the ROS stress in GXO was much milder than that in SJO.

The cell wall is an essential plant cell compartment and plays a role in the plant morphogenesis process, plant environment interactions, cell differentiation, and plant growth^70^. The involvements of cell wall-related genes in plant SI responses have been increasingly reported, and genes coding pectin methylesterases, polygalacturonases, pectate lyases, cellulose synthases and celluloses were frequently identified to be pollination responsive^49,71^. In this study, we identified six “Cell wall organization”-related DEGs between GXO and SJO, and all of them were upregulated in GXO. The differential expression of these cell wall metabolism-related genes in GX cross-pollinated ovaries might contribute significantly to the cell division and ovary development ^72,73^.

### Cross pollination significantly influenced the solute transport in the SJ ovary

Solute transport is an essential biological process in plant cell development^74^. ABC transporters ^75–78^, and WATs have been frequently reported to significantly regulate gametophyte development, seed development, and organ formation by mediating the transportation of phytohormones, such as IAA, GA, and CK^79,80^. Furthermore, SWEETs can promote the long-distance transport of sugar, seed development and fruit development by driving the biosynthesis and transportation of Gas^81–83^. We found that GX cross pollination significantly improved the IAA and GA contents in ovaries, and significantly upregulated the expression of IAA and GA biosynthesis-related genes, such as *GH3.9*, *GA20* and *CPS*. Moreover, some IAA, GA and CK transportation- related genes, such as *ABCG14*, four *WAT1s* and *SWEET3b*, were also significantly up-regulated in GXO. Therefore, Cross pollination can influence the ovary development by regulating the biosynthesis and transportation of phytohormones.

## Conclusions

**Figure 5:**
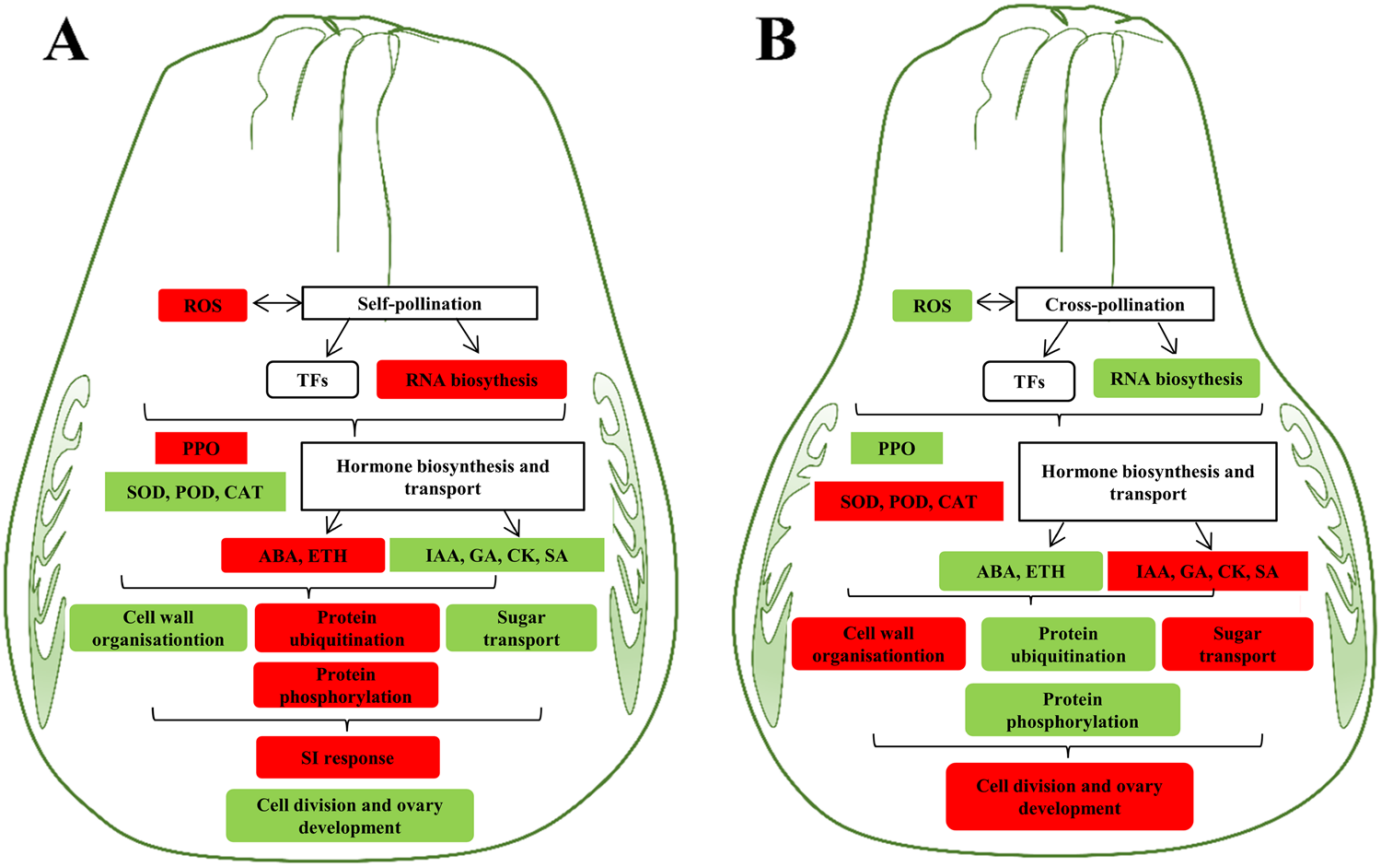
Summary diagram for the physiochemical and transcriptomic differences between self-pollinated (**A**) and cross-pollinated (**B**) ‘Shuijingmiyou’ pummelo ovaries. Cross pollination significantly influenced the pollen tube elongation, fertilization and ovary development of SJ pummelo, which might finally result in improved fruit shape quality. Many physiochemical and transcriptomic differences were found between self- and cross-pollinated ‘Shuijingmiyou’ pummelo ovaries. And the increased and decreased gene expression/ physiochemical parameters/ biological processes were shown in red and green boxes, respectively. ROS: reactive oxygen species; TF: transcription factor; PPO: polyphenol oxidase; SOD: superoxide dismutase; POD: peroxidase; CAT: catalase; Eth: ethylene; IAA: indole acetic acid; CK: cytokinin; GA: gibberellin/ gibberellic acid; SA: salicylic acid; SI: self-incompatibility.

Our study found that GX cross pollination showed the best FS promoting effects on SJ fruits by shortening their fruit neck. Furthermore, comparative physiochemical and transcriptomic analysis of self- and cross-pollinated SJ ovaries revealed that cross pollination using GX pollens strengthened the IAA, GA, and SA accumulation and inhibited the ABA and ethylene biosynthesis in the SJ ovary, which was helpful for the pollen germination, pollen tube development, and ovary development. Moreover, cross pollination suppressed the ROS accumulation and significantly influenced the expression of solute transport, hormone metabolism, cell wall metabolism and RNA biosynthesis-related genes. Based on the morphological, physiochemical, and transcriptomic differences between SJO and GXO, we designed a draft model classifying the influences of cross-pollination on the fertilization and ovary development of ‘Shuijingmiyou’ pummelo (Fig. 5). The results obtained in this study will help understand the influences of cross pollination on pummelo ovary and fruit development and can provide a basis for clarifying the underlying mechanism of cross-pollination improved fruit quality.

### Supplementary Information

Supplementary Figure S1.

Supplementary Table S1.

Supplementary Table S2.

Supplementary Table S3.

Supplementary Table S4.

## Data Availability

All data generated or analyzed in our study are available in this article and its supplementary information files. The transcriptome datasets have been deposited in NCBI (https://www.ncbi.nlm.nih.gov/) with BioProject ID of PRJNA795605 and BioSample accession number of SAMN24737524.
