# Supplementary material for: Comparative physiochemical and transcriptomic analysis reveals the influences of cross-pollination on ovary and fruit development in pummelo (Citrus maxima)
Source: Sci Rep. 2023 Nov 4;13:19081. doi: 10.1038/s41598-023-46058-3 (PMC10625566; doi:10.1038/s41598-023-46058-3)
Supplement: Supplementary file 1 — Supplementary Figure S1. [file 41598_2023_46058_MOESM1_ESM.pdf]

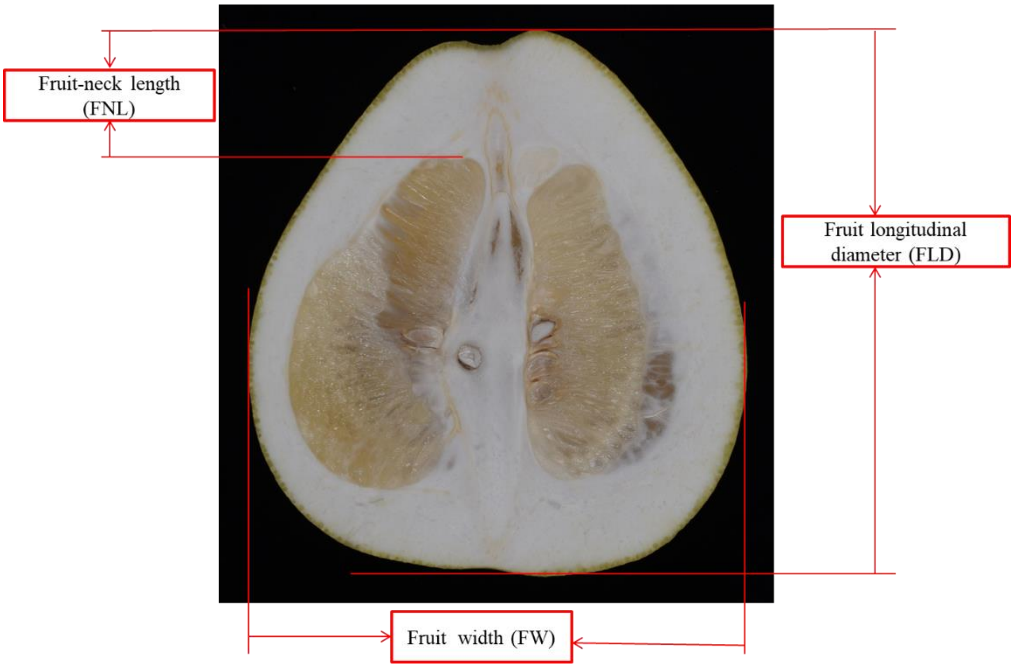

Supplemental Figure S1. Summary diagram for the parameter of fruit shape, FW: Fruit width, FLD: Fruit longitudinal diameter, FNL: Fruit-neck length
